# Performance Evaluation of Cardiac Troponin I Assay: A Comparison Between the Point-of-care Testing Radiometer AQT90 FLEX and the Central Laboratory Siemens Advia Centaur Analyzer

**DOI:** 10.7759/cureus.4231

**Published:** 2019-03-11

**Authors:** Sibtain Ahmed, Lena Jafri, Ahmed Raheem, Shahid Shakeel, Imran Siddiqui

**Affiliations:** 1 Pathology & Laboratory Medicine, Aga Khan University Hospital, Karachi, PAK; 2 Pathology, Aga Khan University Hospital, Karachi, PAK; 3 Pathology & Laboratory Medicine, Aga Khan University, Karachi, PAK

**Keywords:** troponin, point of care test, myocardial infarction, validation, laboratory

## Abstract

Background

To validate the point of care testing (POCT) Trop-I analyzer and compare it with a central laboratory-based chemiluminescence immunoassay, in order to evaluate its performance for use in critical care areas. Moreover, for clinical decision-making, it is imperative to know the extent to which patient stratification will differ based on the analytic method being used. In particular, the aim of this study was to evaluate the analytical performance of the point-of-care analyzer and demonstrate the agreement with the central laboratory measurements in patients presenting to the emergency department (ED) with chest pain and suspected acute coronary syndrome (ACS).

Methods

This cross-sectional study was performed at the section of chemical pathology, department of pathology and laboratory medicine, the Aga Khan University (AKU), Karachi, from October to November 2017. Samples from patients and the quality control material of Trop-I were analyzed for imprecision, linearity, and method comparison on Advia Centaur (Siemens Diagnostics, CA, USA), and the AQT90 FLEX analyzer (Radiometer Medical ApS, Brønshøj, Denmark) with photometric detection at the section of chemical pathology, AKU. Statistical analysis was done using Microsoft Excel (Microsoft Corporation, Washington, United States) and EP Evaluator version 10.3.0.556 (Data Innovations, LLC, VT, US). Quantitative variables were represented in terms of mean ± SD. For precision, the computed SD was compared with allowable random error. Furthermore, Cohen’s kappa was applied to observe the agreement between the two methods.

Results

The Trop-I Precision study on the POCT analyzer showed a coefficient of variation (CV) of 2.4% using a pooled patient sample with a mean Trop-I of 2.15 ± 0.05 ng/ml. Three standards ranging from 0.034 to 1.316 ng/ml were run in triplicate to verify accuracy and linearity. The allowable systematic error (SEa) was 10.0%. The maximum deviation for a mean recovery from 100% was 4.1%. All three of the mean recoveries were accurate and within the allowable error limits. The results were linear with slope 1.04, intercept 0.0. On a method comparison, Trop-I showed good agreement, yielding a kappa value of 0.95.

Conclusion

This study has validated the performance of a POCT Trop-I assay against a central laboratory immunoassay and found acceptable results. POCT assays for cTnI should be implanted in emergency settings to ensure the fast triage of patients with chest pain, as well as timely diagnosis.

## Introduction

The rapid diagnosis of acute coronary syndrome (ACS) relies pivotally on cardiac troponin (cTn) assays [1]. From a laboratorian’s perspective, cTn has been widely accepted as the leading biochemical marker of myocardial injury owing to its exclusive synthesis in the myocardium [2]. As per the recommendations of the American College of Cardiology, the results of cTn should be ideally available within 30 to 60 minutes of patient presentation into critical care areas [3]. However, an evaluation of worldwide practices shows that most of the central hospital laboratories find it difficult to comply with these guidelines of the rapid availability of cTn results for optimum patient management [4].

The possible solution to meet the optimum turnaround times for cTn, especially in emergency settings, is bedside point-of-care systems [5]. The POCT devices offer potential benefits, ranging from the prevention of delays in sample transportation and processing in a central laboratory, and further eradicates interruptions caused by the non-availability of central laboratories for various clinical setups [6].

On the contrary, POCT has certain limitations as well as necessitates rigorous quality control and adequate training of emergency department (ED) end users [7]. Various studies have reported extensive alarms regarding the potentially decreased accuracy of bedside analyzers and have revealed the impact of false negative and false positive results on patient management, unjustified expenditure, and test requests [8-9]. Discordances defined as one method reading at more than the 99th percentile limit and the comparator less than the limit between the cTn-I values among different POCT assays and central laboratory assays have been reported [10-11].

The clinical laboratory, as a standards institute, recommends that laboratories should validate and evaluate the performance of POCT devices with pre-validated central laboratory-based assays to meet acceptable performance standards [12]. Moreover, for clinical decision-making, it is imperative to know the extent to which patient stratification will differ based on the analytic method being used.

In this study, we evaluated two different cTn-I assays, one POCT (AQT90 FLEX, Radiometer Medical ApS, Brønshøj, Denmark) and one used in emergency and routine situations at our laboratory (Advia Centaur, Siemens Diagnostics, CA, USA). In particular, the aim of this study was to evaluate the analytical performance of the point-of-care analyzer and demonstrate the agreement with the central laboratory measurements in patients presenting to the emergency department (ED) with chest pain and suspected ACS.

## Materials and methods

This study was conducted at the section of chemical pathology, department of pathology and laboratory medicine, the Aga Khan University Hospital, Karachi, from October to November 2017. The section of chemical pathology in the central laboratory caters to the emergency department of one of the largest and extensive private sector hospitals in the region.

The cTn-I is analyzed in the central laboratory on the Siemens Adiva Centaur platform. The system was monitored by routine internal quality control procedures per the Clinical and Laboratory Standards Institute (CLSI) standards and participation in College of American Pathologists (CAP) external quality assurance surveys.

Forty de-identified patient samples with cTn-I levels in the range of 0.006 to 50 ng/ml measured on the Siemens Advia centaur chemiluminescence immunoassay collected in the course of routine patient care presenting at the ED with chest pain and ischemic symptoms to rule in or rule out ACS were subjected to testing on the POCT Radiometer AQT90 FLEX analyzer. As the study was non-interventional, informed consent from the participants was not required. Additionally, three standards as provided by the manufacturer were also used to verify the precision accuracy and linearity of cTn-I testing on both analyzers. The diagnostic cutoff for acute coronary syndrome was taken as >0.04 ng/ml for the Advia Centaur and >0.023 ng/ml for the AQT 90 Flex analyzer as recommended by the manufacturer's package insert.

Statistical analysis was done using EP Evaluator version 10.3.0.556 (Data Innovations, LLC, VT, US). Quantitative variables were represented in terms of mean ± SD. For precision, the computed SD was compared with an allowable random error. Furthermore, Cohen’s kappa was applied to observe the agreement between the two methods.

## Results

A total of 40 samples measured on the Siemens Advia Centaur chemiluminescence immunoassay in routine clinical care cTn-I levels were subjected to a re-analysis on the POCT Radiometer AQT90 FLEX analyzer. It was ensured that the cTn-I samples used for the study covered the entire analytical measurement range from 0.006 to 50 ng/ml. Nineteen (47.5%) of the samples had cTn-I above the cut off (>0.04 ng/ml).

**Figure 1:**
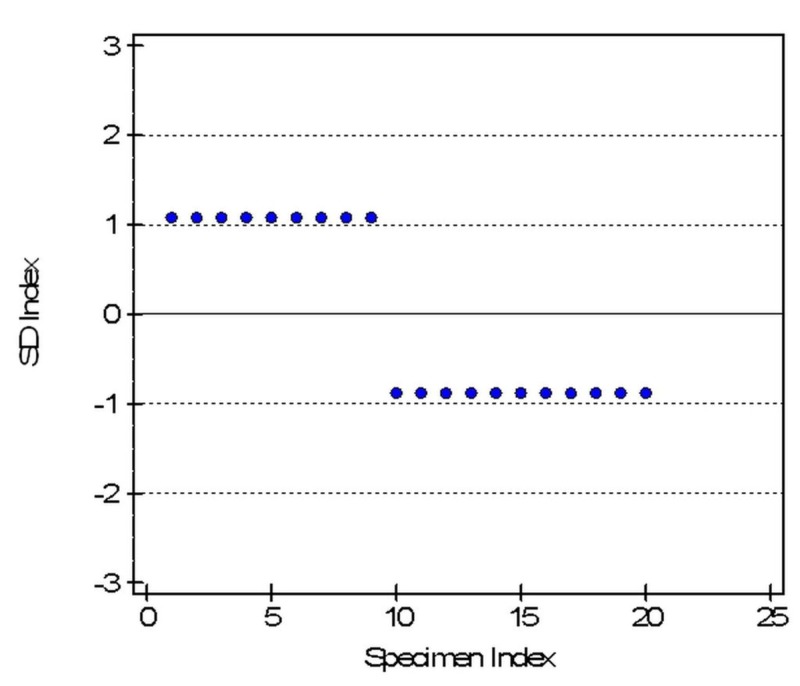
Precision plot for cTn-I analyzed by the AQT90 FLEX analyzer Precision plot AQT90 FLEX analyzer: Radiometer Medical ApS, Brønshøj, Denmark

For precision, 20 samples were analyzed in duplicate on the POCT analyzer and the results were inserted into the EP evaluator, which generated a coefficient of variation (CV) of 2.4% with mean cTn-I of 2.15 ± 0.05 ng/ml. The precision study was acceptable as the computed standard deviation of 0.05 did not exceed the allowable random error of 2.5%, as shown in Figure 1.

**Figure 2:**
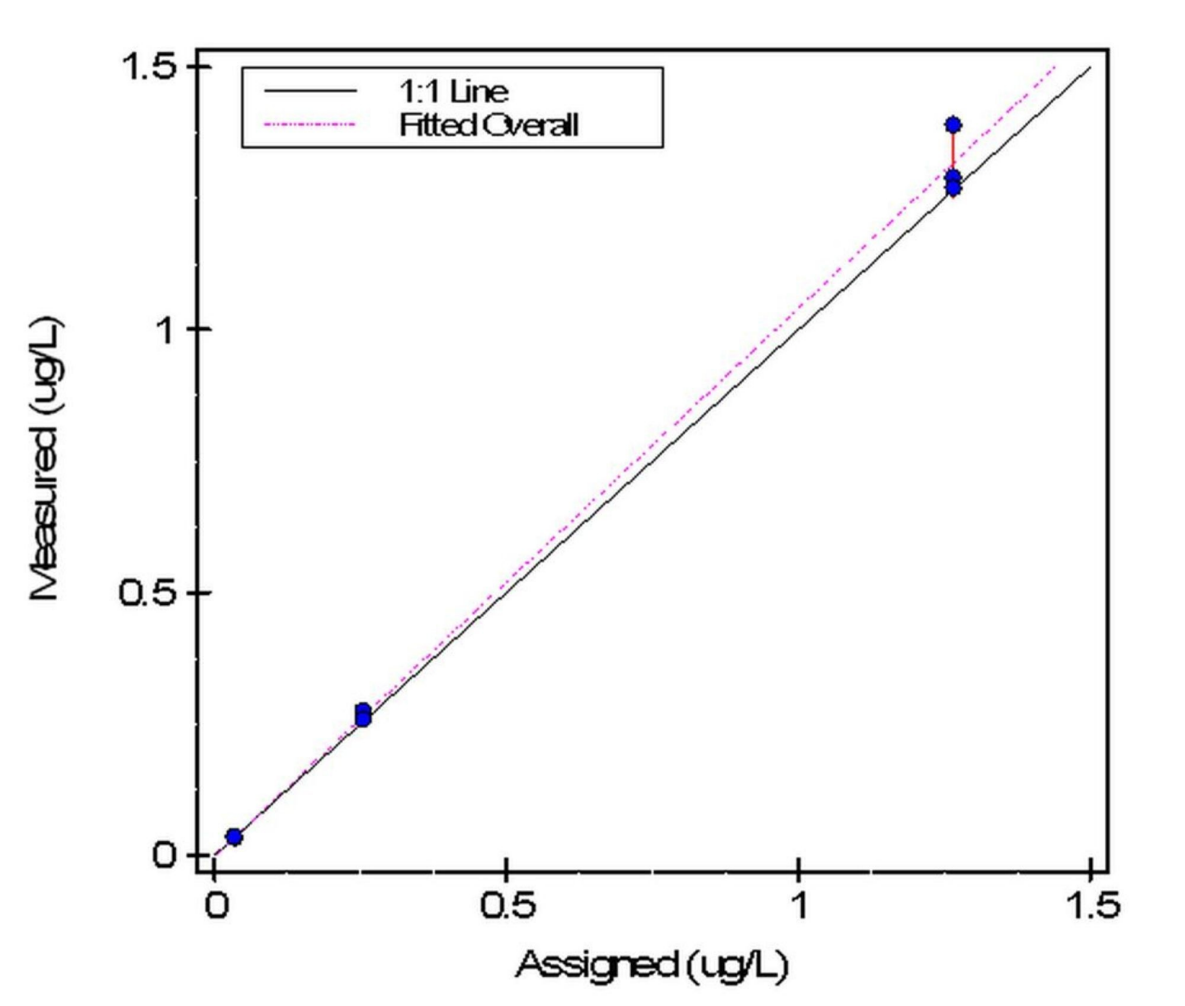
Accuracy plot for cTn-I analyzed by the AQT90 FLEX analyzer Scatter plot

**Figure 3:**
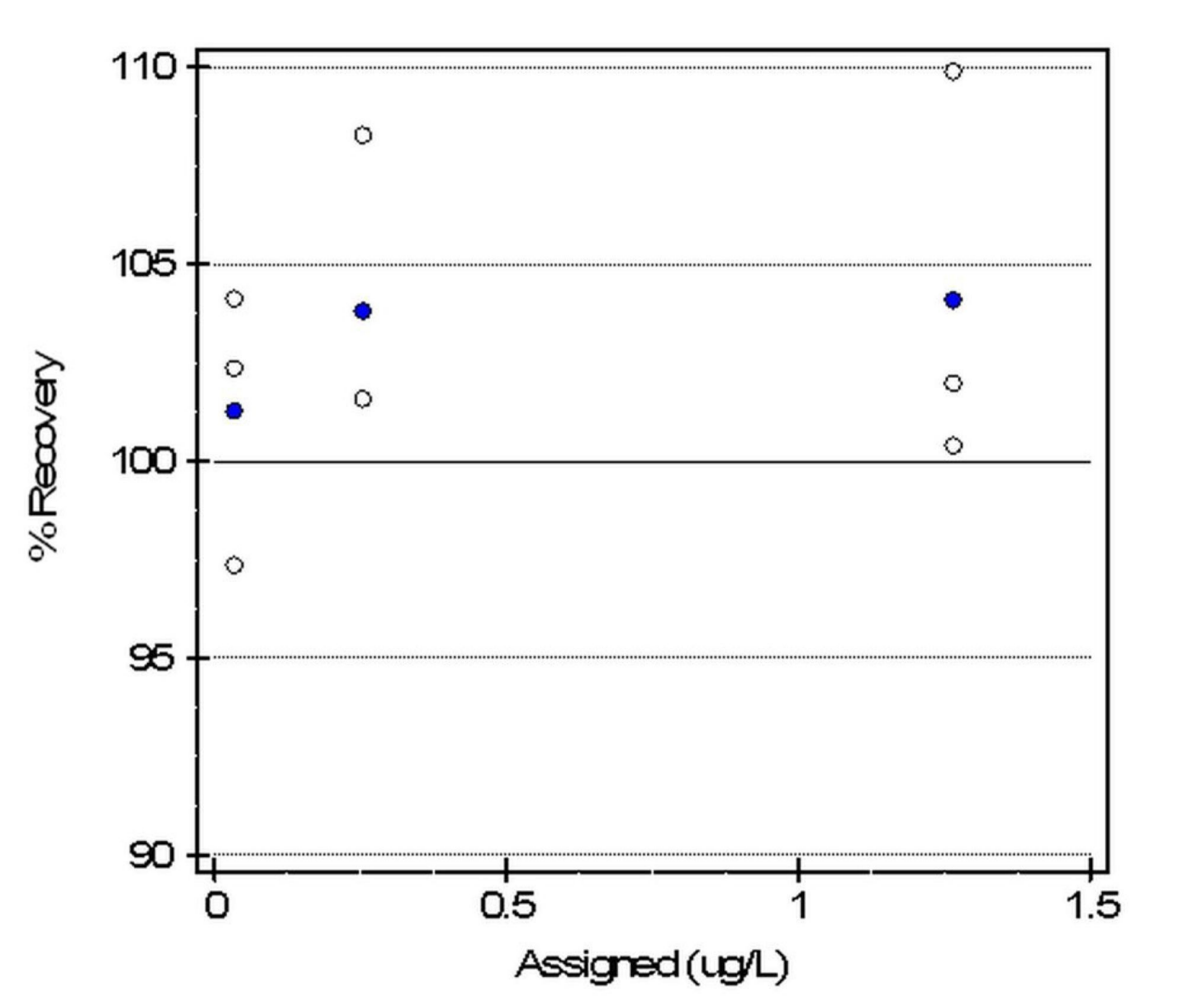
Percent recovery for cTn-I analyzed by the AQT90 FLEX analyzer Percent recovery AQT90 FLEX analyzer: Radiometer Medical ApS, Brønshøj, Denmark

Three standards ranging from 0.034 to 1.316 ng/ml were run in triplicate to verify accuracy and linearity for the AQT 90 FLEX analyzer. The accuracy test was acceptable as the Allowable Systematic error (SEa) was 10.0%. The maximum deviation for a mean recovery from 100% was 4.1%. Three of three mean recoveries were accurate and within the allowable error limits. Furthermore, the results were linear with a slope of 1.04 and intercept 0.0, as demonstrated in Figures 2-3.

For a method comparison between the POCT and central laboratory analyzer, 40 de-identified patient samples re-analyzed on the AQT90 Flex analyzer revealed a good agreement yielding a kappa value of 0.95, as shown in Table 1.

**Table 1:** Agreement between AQT 90 FLEX and Advia Centaur cTn-I AQT90 FLEX analyzer: Radiometer Medical ApS, Brønshøj, Denmark; Advia Centaur: Siemens Diagnostics, CA, USA

	Statistical Summary			
	Negative Reference	Positive Reference	Total	Kappa value
Negative Reference	21(52.5%)	1(2.5%)	22(55%)	0.95
Positive Reference	0(0%)	18(45%)	18(45%)	
Total	21(52.5%)	19(47.5%)	40(100%)	

## Discussion

To the best of our knowledge, this is the first study from Pakistan to evaluate the POCT performance of cTn-I. A significant element in determining the clinical utility of POCT cTn testing is the diagnostic performance of an assay at admission, as compared to a central laboratory assay, to rapidly rule out ACS. Moreover, the importance increases several thousand folds for ED clinical care providers in order to ensure appropriate triage and patient management with the critical goal of improving patient outcomes [13-15].

In 2001, the International Federation of Clinical Chemistry (IFCC) recommended that manufacturers have a target of <10% CV at the myocardial infarction decision limit [16]. A previously undertaken study by Meon et al. and Herbert et al. in a similar setting has reported the acceptable precision of the AQT 90 Flex analyzer considering the stringent <10% CV goal. The results of this study with a CV of 2.4% with mean cTn-I of 2.15 ± 0.05 ng/ml are in concordance with the literature [17-18]. Furthermore, the assay demonstrated acceptable linearity covering a range from 0.034 to 1.316 ng/ml to ensure a reliable clinical utility to rule out or rule in ACS.

For the determination of the clinical utility of POCT cTn-I, 40 patient samples analyzed on the two comparative platforms revealed an agreement of 95%, which is in concordance with other reported literature [19]. Apart from ACS, cTn-I can be elevated in certain non-cardiac pathologies as well as including renal failure [20-21]. In this study, the one patient with a discordant result had chronic renal failure that compromised the performance of the bedside analyzer with a lower cut-off for the diagnosis of ACS compared to Advia Centaur.

The current study has a few limitations. First, the sample size was small, limiting the power of the study and there is a likelihood that the significant variation in findings may arise with larger sample size. Second, this study was not undertaken to associate the utilized cutoffs with clinical diagnosis. Future studies are required in resource-limited set-ups like ours to assess the suitability of POCT testing in clinical settings (e.g., emergency department for strategies to rule in or rule out acute myocardial infarction (AMI)) and cost-effectiveness of standardizing the cTnI cutoffs between POCT and central laboratory assays.

## Conclusions

This study has validated the performance of a POCT Trop-I assay against a central laboratory immunoassay and found acceptable results. POCT assays for cTnI should be implanted in emergency settings to ensure the fast triage of patients with chest pain along with timely diagnosis.
